# Research to inform health systems’ responses to rapid population ageing: a collection of studies funded by the WHO Centre for Health Development in Kobe, Japan

**DOI:** 10.1186/s12961-022-00917-z

**Published:** 2022-11-29

**Authors:** Megumi Rosenberg, Shinichi Tomioka, Sarah Louise Barber

**Affiliations:** WHO Centre for Health Development (WHO Kobe Centre), 1-5-1 Wakinohama-Kaigandori, Chuo-ku, Kobe, 651-0073 Japan

**Keywords:** Population ageing, Older people, Health systems, Research, Universal health coverage

## Abstract

Population ageing is a global phenomenon that has profound implications for all aspects of health systems development. Research is needed to understand and improve the health system response to this demographic shift, especially in low- and middle-income countries where the change is happening rapidly. This Supplement was organized by the WHO Centre for Health Development in Kobe, Japan (WHO Kobe Centre) whose mission is to promote innovation and research for equitable and sustainable universal health coverage considering the impacts of population ageing. The Supplement features 10 papers all based on studies that were funded by the WHO Kobe Centre in recent years. The studies involve a diverse set of 10 countries in the Asia Pacific (Cambodia, Japan, the Lao People’s Democratic Republic, Malaysia, Mongolia, Myanmar, the Philippines, Singapore, Thailand and Viet Nam); address various aspects of the health system including service delivery, workforce development and financing; and utilize a wide range of research methods, including economic modelling, household surveys and intervention evaluations. This introductory article offers a brief description of each study’s methods, key findings and implications. Collectively, the studies demonstrate the potential contribution that health systems research can make toward addressing the challenges of ensuring sustainable universal health coverage even while countries undergo rapid population ageing.

## Background

Population ageing is progressing at a rapid pace worldwide. The share of the global population aged 65 years or older is expected to increase from 9.3% in 2020 to around 16.0% in 2050, surpassing the proportion of children under 5 years (7.1%) and of youth aged 15 to 24 years (13.7%) [1]. It is no longer the case that population ageing and its accompanying health and social challenges are mainly problems for a small handful of economically developed countries. It is clearly a global phenomenon.

Population ageing has profound implications for the progressive achievement of universal health coverage (UHC) in all countries, but especially for the many low- and middle-income countries that will undergo this transition very rapidly [2–5]. As populations age, health systems must adapt to the needs of an older population. This involves a fundamental shift from focusing on acute care and the prevention of premature death to focusing on providing a continuum of care that promotes health across the life course and offers high-quality care, including for older people living with chronic conditions and comorbidities. This requires integrating care across different types and levels of providers and a workforce with the knowledge and skills to address the complex needs of older people [6, 7]. Ensuring access to care for older persons requires not only improving the availability and accessibility of appropriate care but also removing the financial barriers to utilizing care [8]. Systems of financing that protect patients from high out-of-pocket payments are especially important for older people who have greater health care needs [9]. Many countries continue to rely on payroll taxes to finance health care, but population ageing involves a relative reduction in the size of the working-age population. Thus, the way in which health systems are financed is also important to ensure their sustainability and stability in generating revenues for healthcare [10].

Health systems research can provide critical knowledge and evidence to guide health systems reform in countries undergoing population ageing. It can identify the expected impact of population ageing on health systems, the services that are needed and how they should be delivered and resourced. However, such research is still lacking in many low- and middle-income countries.

## Research to accelerate UHC in the context of global population ageing

The WHO Centre for Health Development (WHO Kobe Centre) supports generating new research, building the evidence base and increasing capacity to advance sustainable UHC in the context of global population ageing. As a WHO headquarters department located in Kobe, Japan, the Centre has a unique role in leading this global research agenda while also creating opportunities for the exchange of local and global knowledge.

In July 2017, the WHO Kobe Centre and the Government of Kanagawa Prefecture, Japan, in collaboration with the Ministry of Health, Labour and Welfare, Japan, organized a policy discussion about how to lead health reforms in the twenty-first century, focusing on the themes of UHC, ageing and health systems in countries of the Association of Southeast Asian Nations (ASEAN). It was a follow-up to the ASEAN–Japan Ministerial Meeting on UHC and Population Ageing, which took place during 14–15 July 2017, in which health ministers from ASEAN countries and Japan articulated their commitments to accelerate UHC in light of the challenges of population ageing [11]. Delegates from the organizing bodies and all 10 ASEAN Member States recognized the importance of strengthening the responsiveness of health systems to population ageing to move towards sustainable UHC. They also noted the importance of local researchers in filling the knowledge gaps and guiding countries’ actions. As one of the outcomes of this meeting, the WHO Kobe Centre made a commitment to support a programme of research in ASEAN countries that would generate knowledge about designing the major components of health and social service systems to support ageing populations. The overall purpose of this initiative was to support the generation of new knowledge to inform the development of health systems in ASEAN countries as they move towards implementing sustainable UHC.

By the following month, August 2017, an open call for research proposals was issued by the WHO Kobe Centre, targeting researchers and institutions in ASEAN countries. This special supplement of *Health Research Policy and Systems* presents findings from seven projects that were selected for funding through a competitive process and that involved eight countries (Cambodia, the Lao People’s Democratic Republic [Lao PDR], Malaysia, Myanmar, the Philippines, Singapore, Thailand and Viet Nam). These studies cover a diverse range of issues that are relevant to health systems development when considering population ageing in these countries and other similar contexts.

The studies in Malaysia [12] and Myanmar [13] adapted the survey questionnaire from the Japan Gerontological Evaluation Study [14] to gather basic information about the health and social conditions of the older population, including blood pressure measurements. The two papers from these studies used different analytical approaches, and both found a high prevalence of hypertension and unmet needs for hypertension diagnosis in their populations. Additionally, the study in Malaysia found that blood pressure was not effectively controlled in a majority of those who received antihypertensive medication. These findings strongly suggest there is a need to strengthen the capacity of health systems to prevent, detect and effectively treat hypertension in these and other countries with rapidly ageing populations. The plan in Myanmar is to follow the original cohort to develop a prospective longitudinal study [15].

Similarly, the study in Lao PDR [16] involved a survey of older persons living in the community, and it used an assessment tool developed in another country that had been previously adapted to the Lao context [17]. For the first time, this study has generated data on the prevalence of cognitive impairment among older persons in Lao PDR. The findings showed that more than half of adults older than 60 years had some level of cognitive impairment. This kind of information is critical to understand the growing need for health and social care that comes with population ageing. Yet in many low- and middle-income countries, especially those in the early phases of population ageing, such data and the tools for collecting them are sorely lacking. These studies demonstrate how adapting survey methods and instruments that are well established in other countries can facilitate research on older populations in low- and middle-income countries. The resulting data will be comparable across countries and will allow for exploration of a wide range of health policy, social epidemiological and gerontological research questions.

The studies in Cambodia, the Philippines and Thailand each addressed the supply-side factors of health systems that become increasingly important with population ageing. The study in the Siem Reap Province of Cambodia developed a tool to help staff at primary care-level health centres to assess, counsel, treat and manage patients with hypertension and diabetes [18]. The researchers stressed the importance of ensuring that medication is available and affordable and that primary care centres and higher-level facilities are well integrated to ensure the tool serves its purpose. This tool could be adapted and scaled up for use in other parts of the country. The process could also serve as a model for developing other tools to strengthen primary healthcare services to prevent and control noncommunicable diseases in low- and middle-income countries [19].

The study in the Philippines [20], which involved formative research in both the Philippines and Viet Nam, developed and pilot-tested an in-service, competency-based interprofessional education programme to enhance the capacity of the active workforce to manage the complex health and social care needs of older people through a person-centred approach [21, 22]. An evaluation of this training showed that it successfully increased participants’ positive attitude towards collaborating with geriatric care workers from different professional backgrounds.

The study in Thailand [23] focused on the important role that informal family caregivers play in looking after older people and the need for community-based services to support them, especially in the absence of formal systems of long-term care [24]. The study piloted and rigorously evaluated a novel community-based service model designed to provide respite care, training and support for caregivers, while also offering preventive services to older people. The results showed that the intervention reduced the caregivers’ burden and had positive health effects for the older persons as well.

The innovative solutions developed in these studies and the scientific approaches taken to evaluate them may serve as useful examples for addressing the challenges associated with rapid population ageing faced by the health workforce and for service delivery.

The growing demand for health services arising from population ageing also calls attention to the need for financial protection. The study in Viet Nam revealed new information about the financial impact of out-of-pocket spending on health for older persons [25]. It found that health expenditures related to caring for older persons typically account for most of a household’s expenditures on health and that the risk of incurring a financial burden due to these expenditures was not significantly lower for those with health insurance. The results underscore the need for countries to re-examine the adequacy and appropriateness of financial protection mechanisms, including health insurance benefits, as the population ages.

Many of the studies presented in this supplement faced the challenge of the impacts of the coronavirus disease 2019 (COVID-19) pandemic on research activities in general [26] and, more specifically, on research involving older persons. The original objective of the study in Singapore was to evaluate the effectiveness of a novel care model that incorporates health coaching with integrated health and social care for low-income older populations to reduce hospital readmissions and other unnecessary health visits and to increase competency in self-care. However, due to the significant and prolonged impact of the pandemic, the study could not be completed as planned. Nonetheless, the research team gained valuable experience devising safety-conscious measures and adapting methods of data collection to encourage older people to continue to engage with research during the pandemic. The insights gained from these experiences are shared in a commentary to help ensure that research on vulnerable older populations can be conducted safely and ethically anywhere, whether during the current pandemic or other health emergencies [27].

In addition to these studies in ASEAN Member States, two studies also funded by the WHO Kobe Centre during a similar period are included in this supplement to provide relevant information and evidence. The first is a large-scale study carried out in Kobe City, Japan, that showed how a few simple survey questions designed to assess a person’s cognitive function can be useful both for current assessments and also for predicting their future need for long-term care services [28]. Specifically, a self-reported answer to a question about memory loss that is apparent to one’s family and friends was strongly predictive of the need for long-term care services at 4 years’ followup. The questions used in this survey could be applied to other settings, for example, in Lao PDR as a follow-up to the prevalence study of cognitive impairment to inform planning for long-term care services.

The other is a joint study by the WHO Kobe Centre and the WHO European Observatory for Health Systems and Policies in cooperation with the WHO Regional Office for the Western Pacific that addressed a widely shared concern about the economic impacts of population ageing and the sustainability of public healthcare financing under those conditions [29]. Economic modelling techniques were applied to analyse the effects of age and demographic structure on trends in health expenditures and the moderating effect of improved health in the older working-age population in six countries in WHO’s Western Pacific Region. The paper presented in this supplement describes the simulation results for Mongolia, which suggested that good health at older ages could moderate the potentially negative effects of population ageing on economic growth and health spending [30]. This study sends an important message to policy-makers who are concerned about decreased revenue and increased health expenditures due to population ageing: these consequences are not inevitable, but rather they can be significantly mitigated or altered by making policy choices that enable older people to live in good health.

## Conclusions

The continuing trend towards global population ageing means that health service delivery, financial protection and healthcare financing must account for the complex healthcare needs of older people. The rapid speed at which the population is ageing in many countries calls for an agile response by health systems. Research can play a key role to ensure that this response is well informed. The studies in this supplement present new knowledge and evidence that will be helpful in guiding countries’ actions across different areas of health systems development. They also demonstrate the wide range of methods that can be used to answer research questions and could be feasibly applied to diverse country contexts. The potential value of these experiences and knowledge extends beyond the Asia Pacific region where the studies were implemented. Efforts to support these kinds of research will continue to be critical to ensure that all countries stay on course towards ensuring sustainable UHC even while they undergo this major demographic transition.

## Data Availability

N/A.

## Abbreviations

ASEAN: Association of Southeast Asian Nations
COVID-19: Coronavirus disease 2019
Lao PDR: Lao People’s Democratic Republic
UHC: Universal health coverage
